# Site-specific molecular analysis of the bacteriota on worn spectacles

**DOI:** 10.1038/s41598-020-62186-6

**Published:** 2020-03-27

**Authors:** Birgit Fritz, Melanie März, Severin Weis, Siegfried Wahl, Focke Ziemssen, Markus Egert

**Affiliations:** 1 Faculty of Medical and Life Sciences, Institute of Precision Medicine, Microbiology and Hygiene Group, Furtwangen University, Jakob-Kienzle-Strasse 17, 78054 Villingen-Schwenningen, Germany; 2 Institute for Ophthalmic Research, Eberhard-Karls University, Elfriede-Aulhorn-Straße 7, 72076 Tuebingen, Germany; 3 Center for Ophthalmology, Eberhard-Karls University, Elfriede-Aulhorn-Straße 7, 72076 Tuebingen, Germany

**Keywords:** Microbiology, Microbial communities

## Abstract

Regularly touched surfaces are usually contaminated with microorganisms and might be considered as fomites. The same applies for spectacles, but only little is known about their microbial colonization. Previous cultivation-based analyses from our group revealed a bacterial load strongly dominated by staphylococci. To better account for aerotolerant anaerobes, slow growing and yet-uncultivated bacteria, we performed an optimized 16S rRNA gene sequencing approach targeting the V1-V3 region. 30 spectacles were swab-sampled at three sites, each (nosepads, glasses and earclips). We detected 5232 OTUs affiliated with 19 bacterial phyla and 665 genera. *Actinobacteria* (64%), *Proteobacteria* (22%), *Firmicutes* (7%) and *Bacteroidetes* (5%) were relatively most abundant. At genus level, 13 genera accounted for 84% of the total sequences of all spectacles, having a prevalence of more than 1% relative abundance. *Propionibacterium* (57%), *Corynebacterium* (5%), *Staphylococcus* (4%), *Pseudomonas*, *Sphingomonas* and *Lawsonella* (3%, each) were the dominant genera. Interestingly, bacterial diversity on the glasses was significantly higher compared to nosepads and earclips. Our study represents the first cultivation-independent study of the bacteriota of worn spectacles. Dominated by bacteria of mostly human skin and epithelia origin and clearly including potential pathogens, spectacles may play a role as fomites, especially in clinical environments.

## Introduction

About 48% of all individuals in Europe wear spectacles^1^, i.e. spectacles are remarkably widespread in population. Due to their exposed position in the center of the human face, their close contact to the human skin, nose and mouth, and regular contact with human hands, spectacles are thought to be contaminated with a diverse microbiota. It is well known that surfaces with regular contact to the human body become easily contaminated with microorganisms and consequently can be considered as fomites. The same should apply for spectacles, but only little is known about their microbial load and the hygienic relevance resulting from it.

Previous studies showed that surgeons’ eyeglasses represent fomites in clinical environments^2^. These spectacles were highly contaminated with *Staphylococcus epidermidis*, and it has been suggested that this contamination might represent a risk to patients during operations. Consequently, surgeons were advised to disinfect their spectacles on a regular basis.

To get a first glance into the composition of the spectacle microbiota, we recently performed a cultivation-based study using worn spectacles from university staff and students and from inhabitants of a nursing home for elderly people. We found significant amounts of bacteria on all investigated spectacles and could show that spectacles from elderly people had a more diverse taxonomic composition. Many of the identified bacteria represented potential pathogens that may cause skin and eye diseases^3^. This may be particularly problematic in clinical environments and for infection-susceptible groups of persons, such as immunocompromised or elderly people. We provided a first description of aerobic bacteria on spectacles, but many other groups remained elusive as only (aerobic) cultivation-based methods were used. Clearly, (aerotolerant) anaerobes, slow growing and yet-uncultivated bacteria were probably discriminated against with this approach.

In this study, we examined the bacteriota composition of 30 spectacles at 3 different sample sites (earclips, nosepads and lenses) using Illumina MiSeq-based 16S rRNA amplicon sequencing. All investigated spectacles were obtained from university staff or students. To the best of our knowledge, this is the first molecular study on the bacteriota of spectacles so far. We believe that it provides a solid, cultivation-independent basis for a deeper understanding of the hygienic relevance of these very widespread items that aid human vision.

## Results

### Sequencing results

Out of 5707896 raw sequences, we obtained an average of 22193 operational taxonomic units (OTUs) per sample after quality filtering and deletion of chimeric sequences (11%). After rarefication to 21416 reads per sample and removal of singleton taxa, we identified 5232 OTUs from 85 samples across the three sample sites (28 earclips, 28 nosepads and 29 glasses). The taxonomic assignment of the OTUs revealed 19 bacterial phyla, 52 classes, 105 orders, 241 families and 665 genera. Metadata, original OTU table and taxonomic assignment for all OTUs can be found in the supplementary file 1.

### Diversity analyses

**Figure 1:**
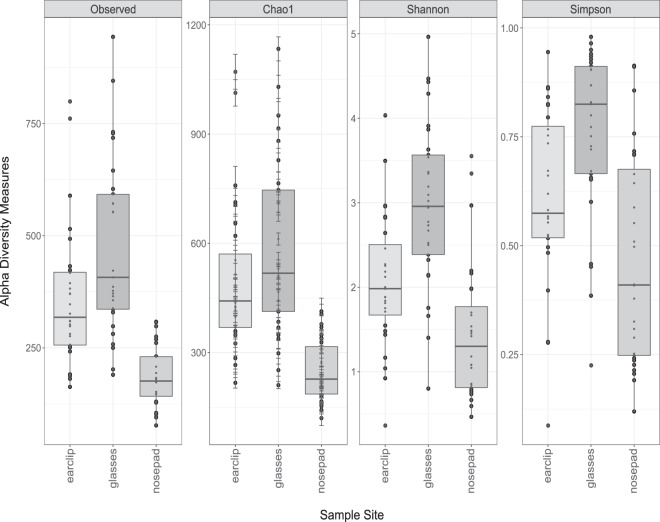
Comparison of alpha diversity measures between the three sample sites (earclips, glasses, nosepads). Differences are shown by four indices (observed taxonomic units, Chao1 estimated species richness, Shannon and Simpson diversity index). All differences were found statistically significant (p < 0.05).

To determine which surfaces hosted the most diverse communities, diversity metrics were calculated. Alpha diversity results revealed more species and a higher diversity on the glasses, compared to nosepads and earclips (Fig. 1). The differences within the factor “sample site” were statistically significant (ANOVA, Analysis of Variance, p < 0.001) for all diversity indices, using Holm-corrected p-values. Therefore, we performed a multiple pairwise comparison between the means of groups applying Tukey HSD (Honestly Significant Difference) as post-hoc test. We found statistically significant differences between the nosepads and glasses for all indices (p < 0.05), as well as significant differences between the nosepads and earclips (p < 0.05) and between glasses and earclips (p < 0.05, except for Chao1 with p > 0.05). Exact (adjusted) p-values can be found in the supplementary file 2 (Statistical analyses performed in R).

**Figure 2:**
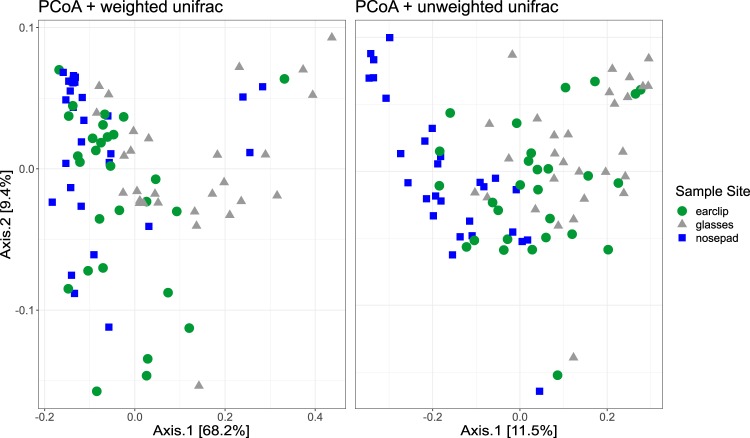
PCoA plots of weighted and unweighted UniFrac distances. Displayed are all samples, assigned on OTU level to the different sample sites (earclips, glasses, nosepads).

To assess beta-diversity, we calculated structural similarity and variation between the microbiota from the sample sites using weighted and unweighted UniFrac-Principal Coordinates Analysis (UniFrac-PCoA) and the UniFrac distance analysis. The PCoA plot of the unweighted UniFrac data suggests that the samples cluster according to glasses and nosepads/earclips. The PCoA plot of weighted UniFrac data rather indicates a clustering of nosepads and glasses/earclips (Fig. 2).

ANOSIM (Analysis of Similarities) on UniFrac distances revealed significant differences in beta diversity between the sample sites (unweighted UniFrac: R = 0.316, p < 0.05; weighted UniFrac: R = 0.161, p < 0.05). By comparing the different sites with each other, ANOSIM on UniFrac distances using Holm-corrected p-values revealed a statistical difference between all the tested sites (adjusted p < 0.05).

As shown in Figs. 1 and 2, spectacle lenses carried the most diverse bacterial community. Exact (adjusted) p-values can be found in the supplementary file 2 (Statistical analyses performed in R).

### Taxonomic composition

**Figure 3:**
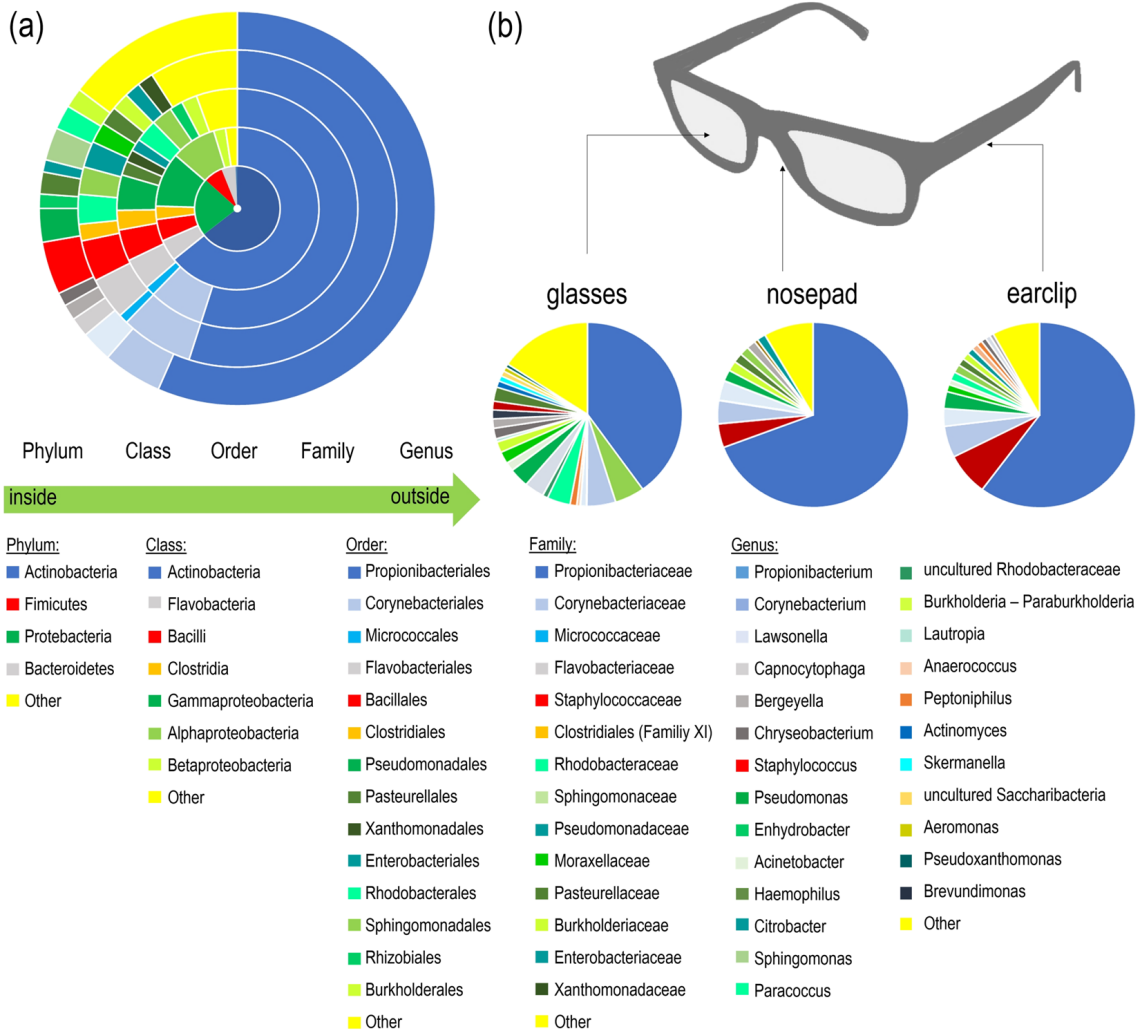
Taxonomic composition of the spectacle bacteriota. (**a**) Multi-level ring chart showing the taxonomic composition of the spectacle bacteriota, as delivered by Illumina-based 16S rRNA amplicon sequencing of 85 samples from 30 spectacles. (**b**) Pie charts showing the taxonomic composition of the different sample sites (earclips, n = 28, nosepads, n = 28, glasses, n = 29). For simplicity, only the taxa with a relative abundance of more than 1% are shown (**a,b**). All taxa with an individual relative abundance of <1% are grouped as “others”. Spectacle graphic art was obtained from https://freesvg.org with a CC0 licence.

The dominant bacterial phyla across all sample sites were *Actinobacteria* (64%), *Proteobacteria* (22%), *Firmicutes* (7%) and *Bacteroidete*s (5%) (Fig. 3a). At genus level, just 14 genera accounted for 85% of the total sequences of all spectacles, with a prevalence of more than 1% relative abundance. The most relatively abundant taxon was *Propionibacterium* with an overall average relative abundance of 57%, followed by *Corynebacterium* (5%), *Staphylococcus* (4%), *Pseudomonas* (3%), *Sphingomonas* (3%), *Lawsonella* (3%), *Paracoccus* (2%), *Haemophilus* (2%), *Burkholderia-Paraburkholderia* (2%) and *Capnoycytophaga* (2%).

Due to the used sequencing technology, it was not possible to analyze complete 16S rRNA genes. Therefore, the taxonomic identification is limited. In order to classify the most abundant OTUs on species level, we performed BLAST (Basic Local Alignment Search Tool) analyses against two databases (Table 1). The majority of the identified taxa belonged to risk group 2 (classification according to German technical rules for biological agents (TRBA) #466)^4^, i.e. represent potential pathogens.

**Table 1:** List of the most abundant OTUs, aligned against two different databases for species affiliation.

OTU	SILVA genus	Relative abundance (%)	BLAST result and accession no.	RG*	Sequence similarity (%)	eHOMD result and accession no.	RG*	Sequence similarity (%)	Origin
*denovo11578*	*Propionibacterium*	55.96	*Cutibacterium acnes* CP033842.1	2	99.8	*Cutibacterium acnes*530_5256	2	99.0	skin
*denovo26270*	*Propionibacterium*	0.82	*Cutibacterium granulosum*LT906441.1	2	99.8	*Cutibacterium granulosum*114N078A	2	99.8	skin
*denovo21272*	*Staphylococcus*	3.63	*Staphylococcus epidermidis* MK542833.1	2	99.8	*Staphylococcus epidermidis*601_3363	2	99.8	skin
*denovo11292*	*Staphylococcus*	0.60	*Staphylococcus hominis MG254773.1*	2	99.8	*Staphylococcus hominis*127N087A	2	99.6	skin/axillae/pubic
*denovo14106*	*Corynebacterium 1*	2.84	*Corynebacterium tuberculostearicum* KJ081533.1	2	99.8	*Corynebacterium tuberculostearicum*077N045A	2	99.8	skin/mucosa
*denovo120*	*Corynebacterium 1*	0.50	*Corynebacterium sp. strain CAU 1475* MG460588.1	ND	99.6	*Corynebacterium coyleae 3*41N142B	2	99.8	environment/mucosa/skin
*denovo16140*	*Lawsonella*	2.76	*Lawsonella clevelandensis* CP009312.1	2	98.5	*Lawsonella clevelandensis*173N105C	2	99.8	environment/skin /not fully defined
*denovo21616*	*Pseudomonas*	1.58	*Pseudomonas guaguanensis* KY471631.1	ND	100	*Pseudomonas pseudoalcaligenes*740_6666	1	98.8	environment/water/not fully defined
*denovo16838*	*Enhydrobacter*	0.84	*Moraxella osloensis* CP024443.2	2	99.8	*Moraxella osloensis* 711_5304	2	99.6	skin/ mucosa /respiratory tract
*denovo4497*	*Haemophilus*	1.12	*Haemophilus parainfluenzae* LT695215.1	2	99.8	*Haemophilus parainfluenzae* 718N000A	2	99.8	nasal/oral
*denovo22674*	*Burkholderia- Paraburkholderia*	1.51	*Burkholderia cenocepacia* EF634151.1	2	100	*Burkholderia cepacia* 571_7530	2	99.4	environment
*denovo893*	*Paracoccus*	0.73	*Paracoccus yeei* CP031078.1	2	100	*Paracoccus yeei* 104N072A	2	100	environment/not fully defined
*denovo16824*	*Capnocytophaga*	0.59	*Capnocytophaga sputigena* CP031078.1	2	99.6	*Capnocytophaga sputigena* 775_4920	2	99.6	oral

The most abundant OTUs were aligned against two different databases (NCBI and eHOMD) to identify the closest known bacterial representative on species level. Only taxa with a relative abundance of >0.5% are displayed. *RG = risk group classification according to German TRBA 466; N/D = no data available.

## Discussion

In this study, we identified the bacteriota on different parts of worn spectacles, their community structure and diversity. We found statistically significant differences within the alpha and beta diversity indices between the three sample sites (nosepads, earclips and glasses). Therefore, it is safe to assume that the sampled site plays a significant role for bacterial community composition. In particular the glasses tended to differ from the other sample sites, as they carried the most diverse bacterial community.

We assume that bacteria are transferred easily from human skin to the earclips and nosepads, whereas glasses are in a more remote position to the skin and exposed to other microbial sources, such as air and dust. Regular cleaning measures^3^ and the lens material, as previously reported for contact lenses^5,6^ or water pipe materials^7^, might also contribute to a different taxonomic composition here. Factors such as the age of the spectacle wearer, gender, material of frames and different lens coatings may influence the bacterial community composition as well^8,9^. We evaluated the influence of age and gender, but for now we could not detect any statistical significant association with alpha and beta diversity. With respect to age, this may be due to the low spread of age data in our subject group. Shibagaki and colleagues^8^ showed a change in community composition of skin bacteria with age, but only between younger (about 30 a old) and older (about 70 a old) subjects.

Regarding the taxonomic composition, we found relatively high shares of propionibacteria at genus level. In 2016, the cutaneous species of the genus *Propionibacterium* were renamed to *Cutibacterium*^10^. In our case, the SILVA database 128 release returned *Propionibacterium*, as identification result, while searching against the NCBI (National Center for Biotechnology Information) and eHOMD (Human Oral Microbiome Database) 16S databases revealed the respective sequences to be affiliated with *Cutibacterium*. Nevertheless, following the SILVA database outputs, the respective data are presented as *Propionibacterium* here.

Clearly, the majority of bacterial microorganisms found on the investigated spectacles seem to originate from human skin. Propionibacteria, corynebacteria and to a lesser extent staphylococci dominate sebaceous sites as found behind the ears (retroauricular crease) and on the sides of the nostrils (ala of nose)^11–13^, where close contact to earclips and nosepads occurs. Propionibacteria, mainly *P. acnes*, are predominant members of the human skin microbiome^14^. This matches our finding that propionibacteria are the most frequent bacteria on worn spectacles, along with corynebacteria. Propionibacteria are aerotolerant anaerobes, reside in pilosebaceous glands, carry a variety of virulence factors, and therefore are involved in diseases, such as Acne vulgaris^15^. These bacteria were also found in infected eyes suffering from endolphtalmitis^16^.

Staphylococci and corynebacteria colonize moist habitats, such as the palms of the hands^15,17^, and might find their way onto spectacles during cleaning or touching these devices. Previous cultivation-based analyses from our group revealed *S. epidermidis* as the most frequent bacterium on worn spectacles^3^. It is known that *S. epidermidis* normally colonizes human skin without being harmful, but rather being benign or mutualistic for its host^16^. However, many staphylococci include antibiotic resistant strains^18^, such as MRSA (Methicillin-resistant *Staphylococcus aureus*) or MRSE (Methicillin-resistant *Staphylococcus epidermidis)*. Although the relative abundance of staphylococci on spectacles might be lower than previously expected from our cultivation-based study^3^, further investigations should nevertheless examine spectacles as potential carriers of antibiotic resistant bacteria in more detail. This issue could be of high hygienic relevance, especially in clinical environments.

The genus *Pseudomonas* also comprises many species known to cause opportunistic (skin-) infections, and has the potential to be problematic, particularly in clinical environments. Specifically, *P. aeruginosa* can cause severe infections at different anatomic sites^19^. With *Lawsonella*, we identified a just recently described novel genus. BLAST analysis revealed our sequences to represent *Lawsonella clevelandensis* (Table 1), presumably an anaerobic bacterium, affiliated with the genus *Corynebacterium*, which are typical colonizers of sebaceous skin^20^. *L. clevenlandensis* was first isolated from human abscesses, mainly from immunocompromised patients. The authors assumed this bacterium to be of environmental origin or as a member of the human skin microbiota and as a potential pathogen^21,22^. Escapa and colleagues^23^ described *L. clevelandensis* to be rather common on oily skin sites, particularly at the alar crease, the glabella and occiput, but also to be present in human nostrils. Apparently, it also occurs on spectacles in rather high shares.

Figure 3 indicates that bacterial genera from the human aerodigestive tract were also frequently detected on the investigated spectacles, such as *Moraxella, Haemophilus, Actinomyces, Capnocytophaga* or *Lautropia*^24–27^. Coughing, sneezing or cleaning with clothes after breathing on the lenses might promote this diversity. Other bacteria that were identified on the spectacles represent ubiquitous or environmental taxa, such as *Skermanella, Paracoccus* or *Aeronmonas*. Notably, many of the identified genera contain species known to cause skin and eye diseases^20,28–30^.

Additional database searches (Table 1) classified the most abundant OTUs on species level. OTUs classified as *Enhydrobacter* rather seem to be affiliated with *Moraxella osloensis*. which is a member of the respiratory tract and nasophyryngeal microbiota that is also known to cause malodor on washed laundry^24,31,32^. The majority of the taxa displayed in Table 1 belong to risk group 2, i.e. they represent potential pathogens. They may be harmless to healthy people but may cause infections in newborns, immunocompromised patients, pregnant women or elderly persons.

## Conclusions and Outlook

Spectacles - widely used devices that aid human vision - carry a significant and highly diverse bacterial load. Our study provides first, cultivation-independent insights into this spectacle bacteriota, which is mainly comprised of bacteria of human skin and epithelia origin. The community was dominated by bacteria typical for the skin areas that are in physical contact with the spectacle frames. The bacteriota on the lenses differed significantly from the other sample sites and showed the highest diversity. As many of the identified genera comprise potentially pathogenic species that may cause skin and eye diseases, spectacles clearly must be regarded as fomites. This is of particular importance in clinical environments, but also for people daily working with worn spectacles, such as opticians.

Future studies should address the role spectacles play as fomites in more detail, e.g. regarding the role as carriers and vectors of multi-resistant bacteria in clinical environments or as reservoirs for microorganisms that can cause recurring eye [space] infections. Clearly, such investigation should also consider less easy accessible parts of spectacles, such as the hinges. Due to the use of *Bacteria*-specific primers we could not detect any fungal or viral species on the spectacles investigated here. However, this would be of additional interest, as there are several fungal and viral taxa known to be involved in severe eye infections^33–35^, such as *Candida albicans, Fusarium solani*, *Aspergillus flavus*, Herpes simplex and Varizella zoster.

In addition, the protocols and data published here might serve as a basis to study the surfaces of other devices with close contact to human eyes and facial skin, such as microscopes, slit lamps or surgeon´s eyeglasses, in order to gain a deeper understanding of their hygienic relevance, too. Finally, the bacterial taxa identified here as being prominent on spectacles might serve as practically very relevant organisms for the testing of antimicrobial coatings and/or cleaning strategies for spectacles.

## Material and methods

### Ethics statement

In this study, no human samples but swab samples obtained from worn spectacles were investigated. All swab samples were collected at Furtwangen University. Spectacles and usage data of the spectacle wearers were provided voluntarily. Informed consent to use the obtained data for scientific purposes was obtained orally. Personal data of the participants were not recorded, rendering it impossible to assign a spectacle microbiota to a specific wearer. Moreover, the spectacle wearers neither provided directly health-related data, nor were the analyses aimed at detecting directly health-related bacteria, such as obligate pathogens. Therefore, we believe that the study was performed in an ethically appropriate manner.

### Spectacle sampling

Spectacles for swab-sampling were kindly provided by 30 students and employees (mean age 24 ± 6.6 years, (mean ± SD), 12 males and 18 females) of Furtwangen University, Campus Villingen-Schwenningen. All collected metadata, such as age, gender or frame material are included in supplementary file 1.

Standardized sampling was performed from October to December 2018 in a university laboratory. Each spectacle was sampled in a meandering pattern, at 3 sites each: lenses (left and right, front and back, respectively), ear clips (left and right side, inside and outside, respectively) and nose pads. One swab sample was obtained per sampled site using dry, sterile Puritan Hydra Flock Swabs (Puritan Diagnostics LLC, Maine, USA). Swabs were broken off into sterile 1.5 ml microfuge tubes, stored at −20 °C, and processed within one week.

### DNA extraction

DNA was extracted and purified from the swab heads using the PureLink Microbiome DNA Purification Kit (Life Technologies GmbH, Darmstadt, Germany) with slight modifications to the manufacturer’s ‘buccal, vaginal or skin swab samples’ protocol. Samples were incubated at 75 °C for 10 min at 850 rpm, followed by five rounds of bead beating in a FastPrep 24 instrument (MP Biomedicals LCC, Santa Ana, USA) for 1 min at 6.5 m/s and then placed on ice for 1 min. After 2 min of incubation at room temperature, the DNA was eluted with 40 µl of elution buffer. The flow through was reloaded onto the same filter, and again incubated for 2 min. After centrifugation, additional 10 µl of elution buffer was added onto the same filter, incubated for 1 min and centrifuged. The purified DNA was stored at −20 °C until further analyses.

### Library preparation

The V1 and V3 hypervariable regions of the bacterial 16S rRNA gene were amplified using the primer pair 63F (5′-CAGGCCTAACACATGCAAGTC-3′)^36^ and 511R (5′-GCGGCTGCTGGCACRKAGT-3′)^37^ (Eurofins Genomics GmbH, Ebersberg, Germany), added to an overhang adapter sequence tail (5′-TCGTCGGCAGCGTCAGATGTGTATAAGAGACAG-3′), yielding a PCR product of ~545 bp. The V1–V3 primer pair covers a typical region widely used for skin microbiome studies^38^, but it’s also recommended for nasopharyngeal areas^39^. We assume, that this region provides an accurate insight into the human skin and nasopharyngeal microbiota, which we expected to dominate on spectacles.

All extracted samples were amplified in duplicates. PCR setup and cycling conditions for the primary amplification were as follows: 3 µl of template DNA, 15.05 µl of nuclease and DNA free water (VWR International, Darmstadt, Germany), 5 µl of 5 × KAPA High Fidelity Buffer (KAPA Biosystems, Wilmington, USA), 0.6 µl of 10 mM KAPA dNTP Mix, 0.25 µl of 20 mg/ml BSA (Life Technologies GmbH), 0.5 µl of KAPA High Fidelity Hot Start Polymerase, 0.3 µl of forward (10 µM) and 0.3 µl of reverse primer (10 µM).

The PCR profile was as follows: 98 °C initial denaturation for 3 min, followed by 35 cycles of 98 °C for 30 s, 63 °C for 30 s, 72 °C for 60 s, and a final extension at 72 °C for 2 min. PCR products were verified by standard 0.8% agarose gel electrophoresis using Midori Green as DNA-dye (Biozym, Olderndorf, Germany). With each batch, water template control reactions were included. As additional negative controls, sterile, unused swabs were prepared as described above. No PCR background contamination from either reagents and/or collection procedures was discovered. As positive controls, we used diluted (1:1000) DNA from overnight cultures of *Escherichia coli* K12, extracted with the same DNA purification kit.

Two replicates of each sample were pooled and cleaned up using Agencourt AMPure XP Beads (BeckmanCoulter Inc., Krefeld, Germany) according to the Illumina library preparation protocol with an adapted bead to sample ratio of 0.7 : 1^40^.

Subsequently, a second amplification step was carried out to anneal dual-index barcodes. The Illumina Nextera XT Index Kit v3 and Nextera XT Index Kit v2 Set B adapters (Illumina Inc., San Diego, USA) with different dual indices were combined to allow multiplexing and good performance of all samples. Two unique indices were attached to each amplicon sample, while performing a second PCR reaction.

We used 5 µl of cleaned amplicon PCR product, with 4 µl index primer i7xxx and 4 µl index primer i5xxx, respectively. 25.6 µl of nuclease and DNA free water, 10 µl of 5 × KAPA High Fidelity Buffer (KAPA Biosystems), 1.2 µl of dNTP Mix (10 mM) and 0.2 µl of KAPA High Fidelity Hot Start DNA Polymerase were added. Cycling started at 98 °C initial denaturation for 3 min, followed by 8 cycles of 98 °C for 30 s, 55 °C for 30 s, 72 °C for 30 s and a final extension at 72 °C for 5 min. Index PCR products were verified by standard 0.8% agarose gel electrophoresis and cleaned up as described above, with a bead to sample ratio of 0.8 : 1. Post PCR quality checks on a Bioanalyzer 2100 Instrument with the DNA High Sensitivity Kit (both Agilent Technologies Deutschland GmbH, Waldbronn, Germany) revealed the exact amplicon size (bp) of each sample. After quantification using a Qubit 2.0 Fluorometer (Life Technologies GmbH), equimolar concentrations were calculated.

### Sequencing

The library was adjusted to 3 nM (with 10 mM Tris buffer, pH 8.5), pooled, combined with 30% PhiX control (Illumina Inc.) and finally diluted to 5 pM. The sequencing was run on an Illumina MiSeq platform using the MiSeq Reagent Kit v3 (600 cycle) (Illumina Inc.) with a quality score ≥30 and default settings. Sequence files were deposited at the European Nucleotide Archive (ENA) under the accession number PRJEB32211.

### Bioinformatics

Sequences were processed in QIIME 1.9.1^41^. Paired end reads were joined using the “join_paired_ends.py” script with default settings. Chimeras from the demultiplexed sequences were removed using “vsearch”^42^. Operational Taxonomic Units (OTUs) were clustered de novo with a 97% similarity threshold using “uclust”^43^. Taxonomy was assigned to representative OTUs against the SILVA database, release 128^44^. Parallel sequence alignment was performed via PyNAST^45^. Chloroplast and mitochondrial OTUs were removed.

To identify the relatively most abundant genera down to species level, their 16S rRNA amplicon sequences were aligned against two different databases to identify the closest known bacterial representative using the standard (nucleotide) BLAST at NCBI (National Center for Biotechnology Information) and eHOMD (Human Oral Microbiome Database; www.ehomd.org)^23^. eHOMD is a database providing 16S rRNA gene sequences from different body sites, especially the human aerodigestive tract. The metadata, the unrarefied OTU table and the taxonomic assignments are provided in the supplementary file 1.

### Statistical analyses

All statistical analyses and graphical visualizations were performed in R 3.5.3 using the “phyloseq”^46^, “vegan”^47^, “coin”^48^ and “microbiome”^49^ packages. Figures were created in R using “ggplot2”^50^ and MS Excel 2016. The analysis-report was created with R-studio (version 1.1.463)^51^ and can be found in the supplementary file 2.

We only kept taxa with a prevalence of more than one. The 85 samples were rarefied to a level of 21416 sequences for even sampling depth (seed: 1121983).

To determine which surfaces hosted the most diverse communities, alpha diversity metrics (Observed, Chao1, Shannon and Simpson) were calculated. For comparative analysis of the diversity indices among the different factors (e.g. sample site), one-way ANOVAs (Analysis of Variance) with Holm-adjusted p-values were performed^52^. For factor-specific categories, pairwise multiple comparisons between the sample sites were calculated using Tukey’s honest significant differences (HSD) as post-hoc test^53^.

In order to measure beta diversity, principle coordinate analysis ordinations (PCoA) were generated based on weighted and unweighted UniFrac distance matrix^54^ while using abundance information of OTUs and phylogeny.

ANOSIM (Analysis of Similarities) calculations on UniFrac distance matrices, using 9999 permutations, were performed as non-parametric tests for similarity between groups using the “vegan” package, version 2.5–5.The ANOSIM statistic variable R ranges from −1 to +1 with a value of 0 indicating no difference between the groups^55^.

All tests were two-sided, and p-values, respectively Holm-adjusted p-values below 0.05 were regarded as statistically significant.

## Supplementary information

Supplementary information

Supplementary information2.

## Data Availability

The sequences supporting the conclusions of this article are available at the European Nucleotide Archive (ENA - https://www.ebi.ac.uk/ena) under the accession number PRJEB32211. All data generated or analysed during this study are included in this published article (and its Supplementary Information files). A full record of all statistical analysis is included as supplementary file 2 and was created using the knitr package in R^56^.
