# Supplementary material for: Site-specific molecular analysis of the bacteriota on worn spectacles
Source: Sci Rep. 2020 Mar 27;10:5577. doi: 10.1038/s41598-020-62186-6 (PMC7101307; doi:10.1038/s41598-020-62186-6)
Supplement: Supplementary file 2 — Supplementary information2. [file 41598_2020_62186_MOESM2_ESM.pdf]

## “Site-specific molecular Analysis of the bacteriota on worn spectacles”

Birgit Fritz, Melanie März, Severin Weis, Siegfried Wahl, Focke Ziemssen, Markus Egert

## Supplementary file 2: Statistical analyses performed in R

## Data

This document contains all statistical analyses conducted for the manuscript. Note that due to the randomiterative nature of some analyses (such as beta-rarefaction, etc.) some of the figure parameters will change slightly during reanalysis, though core results will remain essentially unchanged. All data to reproduce the analysis can be found here: European Nucleotide Archive ENA (https://www.ebi.ac.uk/ena) under the accession number PRJEB32211

## Load necessary analysis libraries

```
library("phyloseq")
library("ggplot2")

## Warning: package 'ggplot2' was built under R version 3.5.3

library("vegan")

## Warning: package 'vegan' was built under R version 3.5.3

## Loading required package: permute

## Warning: package 'permute' was built under R version 3.5.3

## Loading required package: lattice

## This is vegan 2.5-5

library("coin")

## Warning: package 'coin' was built under R version 3.5.3

## Loading required package: survival

## Warning: package 'survival' was built under R version 3.5.3

library("ape")

## Warning: package 'ape' was built under R version 3.5.3
```

## Import data from QIIME (1.9.1)

*L_otu_table_sort_taxafilt.biom* was processed from sequencing data: Join paired ends, split libraries, delete chimeras, otu-picking de novo, pick a representative sequence, assign taxonomy, align sequences, filter alignment, make a phylogenetic tree, make otu-table, sorts otus, remove mitochondria and chloroplasts.

```
biomfile           <- import_biom("L_otu_table_sort_taxafilt.biom")

mapfile            <- import_qiime_sample_data("mapping.txt")

treefile           <- import_qiime(treefilename = "I_rep_set_tre.tre")

## Processing phylogenetic tree...
##  I_rep_set_tre.tre ...

phylotable_otu     <- merge_phyloseq(biomfile,mapfile,treefile)

colnames(tax_table(phylotable_otu)) <- c("Kingdom","Phylum","Class","Order","
Family","Genus","Species")
```

Some important metrics from raw phylotable_otu:

- Min. number of sequences = 23175
- Max. number of sequences = 156419
- Total number of sequences = 5707896
- Number of samples = 85
- Number of OTUs = 22193

## Remove singleton taxa: Keep only taxa with a prevalence of more than one

```
phylotable_excl    <- filter_taxa(phylotable_otu, function (x) {sum(x > 0) > 1
}, prune=TRUE)
```

some important metrics after removal of singletions (phylotable_excl):

- Min. number of sequences = 21416
- Total number of sequences = 5640153
- Number of samples = 85
- Number of OTUs = 6047

## Rarefy the dataset to a minimum number of sequences (21416); set rng seed to 1121983

```
phylotable_rare_otu <- rarefy_even_depth(phylotable_excl, rngseed = 1121983)

## `set.seed(1121983)` was used to initialize repeatable random subsampling.

## Please record this for your records so others can reproduce.

## Try `set.seed(1121983); .Random.seed` for the full vector

## ...

## 815OTUs were removed because they are no longer 
## present in any sample after random subsampling

## ...
```

After rarefication some important metrices

- Number of samples = 85
- Number of OTUs = 5232
- phyla = 19
- order = 105
- families = 241
- genera = 665

## Results

Identify the taxonomic composition on spectacles and their relative abundances.

1.) Calculate abundances on the phylogenetic levels (here calculation only for the phylum level is displayed, for the other levels calculation is executed in the same manner)

```
rank_names(phylotable_rare_otu)

## [1] "Kingdom" "Phylum"  "Class"   "Order"   "Family"  "Genus"   "Species"

phylotable_rare_phy   <- tax_glom(phylotable_rare_otu, "Phylum")

abundances_phyla      <- numeric(nrow(otu_table(phylotable_rare_phy)))

for(i in 1:nrow(otu_table(phylotable_rare_phy))){
abundances_phyla[i] <- as.vector(sum(otu_table(phylotable_rare_phy)[i,])/sum(
otu_table(phylotable_rare_phy)))
 }

matrix_abund_phyla      <- matrix(tax_table(phylotable_rare_phy), nrow = nrow
(tax_table(phylotable_rare_phy)), ncol = ncol(tax_table(phylotable_rare_phy))
)
```

```
row.names(matrix_abund_phyla)   <- row.names(phylotable_rare_phy)
colnames(matrix_abund_phyla)    <- colnames(tax_table(phylotable_rare_phy))

df_abund_phyla          <- cbind(matrix_abund_phyla, "Abundances" = abundance
s_phyla)

write.csv(df_abund_phyla, "abundances_phyla_sort.csv")
```

2. ) Calculate abundances at the different sample sites (caclulation for “Earclip” is displayed, for the other sample sites calculation is executed in the same way):

```
phylotable_earclip      <- subset_samples(phylotable_rare_otu, SampleSite ==
"earclip")
phylotable_earclip_phy  <-   tax_glom(phylotable_earclip, "Phylum")
```

Abundances are calculated as described above.

## Alpha Diversity

Are there any differencs between factors within a sample? What is there and how much?

```
alpha.diversity   <-estimate_richness(phylotable_rare_otu, measures = c("Obse
rved", "Chao1", "Shannon", "Simpson"))
alphadat          <- cbind(sample_data(phylotable_rare_otu), alpha.diversity)
```

For the factor sample site:

```
rare.anovaO        <- aov(Observed ~ SampleSite, alphadat)
rare.anovaO.sum    <- summary(rare.anovaO)[[1]][["Pr(>F)"]][1]

rare.anovaCh       <- aov(Chao1 ~ SampleSite, alphadat)
rare.anovaCh.sum   <- summary(rare.anovaCh)[[1]][["Pr(>F)"]][1]

rare.anovaSh       <- aov(Shannon ~ SampleSite, alphadat)
rare.anovaSh.sum   <- summary(rare.anovaSh)[[1]][["Pr(>F)"]][1]

rare.anovaSi       <- aov(Simpson ~ SampleSite, alphadat)
rare.anovaSi.sum   <-  summary(rare.anovaSi)[[1]][["Pr(>F)"]][1]

rare.anovaTableSa <- c( rare.anovaO.sum,  rare.anovaCh.sum, rare.anovaSh.sum,
rare.anovaSi.sum)
names(rare.anovaTableSa) <- c("Observed", "Chao1", "Shannon", "Simpson")

rare.anovaTableSa

##     Observed         Chao1            Shannon          Simpson
##    1.789361e-09     8.730290e-09     1.421329e-08     7.566388e-06
```

We performed a Holm p-value correction

```
p.adjust(rare.anovaTableSa, method = "holm")

##     Observed        Chao1             Shannon           Simpson
## 7  .157445e-09       2.619087e-08      2.842658e-08      7.566388e-06
```

**Summary of ANOVA p-values for the factor “Sample Site”:**

| | | | |
|---|---|---|---|
| Observed: | p=1,789361e$^{-09}$ | Observed – Holm-corrected: | p= 7.157445e$^{-09}$ |
| Chao1: | p=8,730290e$^{-09}$ | Chao1– Holm-corrected: | p=2.619087e$^{-08}$ |
| Shannon: | p=1.421329e$^{-08}$ | Shannon– Holm-corrected: | p=2.842658e$^{-08}$ |
| Simpson: | p=7.566388e$^{-06}$ | Simpson– Holm-corrected: | p=7.566388e$^{-06}$ |

## Plot Diversity

```
p     <- plot_richness(phylotable_rare_otu, x="SampleSite", measures=c("Obser
ved", "Chao1", "Shannon", "Simpson")) +

theme_bw()

p1    <- p +
theme(axis.text.x=element_text(size=12),axis.text.y  =element_text(size=10))+
xlab("\nSample Site")+
ylab("Alpha Diversity Measures\n") +
geom_boxplot(aes(fill = SampleSite, colour=SampleSite), alpha=0.4) +
theme(axis.title.x = element_text(size =10)) +
theme(axis.title.y = element_text(size =10))

p_diversity <- p1 +
scale_fill_manual("SampleSite", values = c("earclip"= "grey69", "nosepad" = "
grey51", "glasses" = "grey27" )) +
scale_colour_manual("SampleSite", values = c("earclip"= "grey39", "nosepad" =
"grey39", "glasses" = "grey39")) +
geom_point(aes(colour=SampleSite)) +
geom_errorbar(aes(colour=SampleSite, ymin=value-se, ymax=value+se), width=0.1
) +
theme(legend.position="none") +
theme(strip.text.x = element_text(size=10))+
theme(axis.text.x = element_text(angle=90, hjust=1))

plot(p_diversity)

## Warning: Removed 255 rows containing missing values (geom_errorbar).
## Warning: Removed 255 rows containing missing values (geom_errorbar).
```

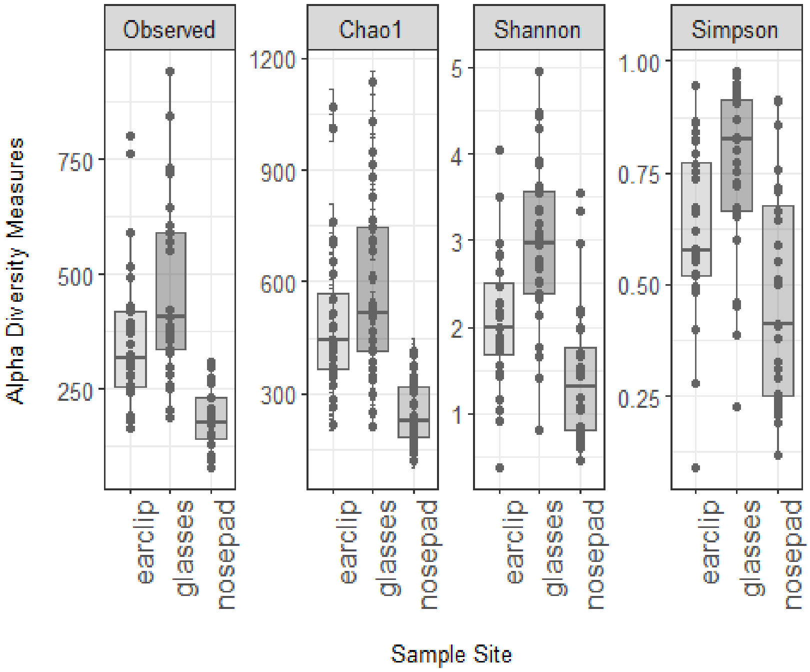


As the ANOVA test is significant, we can compute Tukey HSD (Tukey Honest Significant Differences) to perform paarwise comparisons between the sample sites.

```
TukeyHSD(rare.anovaO) #Observed

##   Tukey multiple comparisons of means
##     95% family-wise confidence level
##
## Fit: aov(formula = Observed ~ SampleSite, data = alphadat)
##
## $SampleSite
##                        diff        lwr        upr        p adj
## glasses-earclip      108.3966   12.99577    203.79733   0.0219609
## nosepad-earclip     -177.5000 -273.73399    -81.26601   0.0000941
## nosepad-glasses     -285.8966 -381.29733   -190.49577   0.0000000

TukeyHSD(rare.anovaSh) #Shannon

##   Tukey multiple comparisons of means
##     95% family-wise confidence level
##
## Fit: aov(formula = Shannon ~ SampleSite, data = alphadat)
##
## $SampleSite
##                         diff        lwr         upr        p adj
## glasses-earclip      0.9127653   0.3672238   1.45830678  0.0004112
## nosepad-earclip     -0.6161477  -1.1664538  -0.06584155  0.0243829
## nosepad-glasses     -1.5289130  -2.0744545  -0.98337149  0.0000000
```

```
TukeyHSD(rare.anovaCh) #Chao1

##   Tukey multiple comparisons of means
##     95% family-wise confidence level
##
## Fit: aov(formula = Chao1 ~ SampleSite, data = alphadat)
##
## $SampleSite
##                         diff        lwr         upr         p adj
## glasses-earclip      89.3419     -31.24998   209.9338    0.1866316
## nosepad-earclip      -245.8036   -367.44869  -124.1585   0.0000190
## nosepad-glasses      -335.1455   -455.73736  -214.5536   0.0000000
```

```
TukeyHSD(rare.anovaSi) #Simpson

##   Tukey multiple comparisons of means
##     95% family-wise confidence level
##
## Fit: aov(formula = Simpson ~ SampleSite, data = alphadat)
##
## $SampleSite
##                          diff         lwr          upr       p adj
## glasses-earclip      0.1527924    0.01835543  0.287229285 0.0219174
## nosepad-earclip      -0.1414599  -0.27707096 -0.005848819 0.0388906
## nosepad-glasses      -0.2942522  -0.42868918 -0.159815320 0.0000039
```

**Summary of the Tukey HSD p-values:**

| | | |
|---|---|---|
| Observed: | glasses-earclip | p = 0.0219609 |
| | nosepad-earclip | p = 0.0000941 |
| | nosepad-glasses | p = 0.0000000 |
| Chao1: | glasses-earclip | p = 0.1866316 |
| | nosepad-earclip | p = 0.0000190 |
| | nosepad-glasses | p = 0.0000000 |
| Shannon: | glasses-earclip | p = 0.0004112 |
| | nosepad-earclip | p = 0.0243829 |
| | nosepad-glasses | p = 0.0000000 |
| Simpson: | glasses-earclip | p = 0.0219174 |
| | nosepad-earclip | p = 0.0388906 |
| | nosepad-glasses | p = 0.0000039 |

## Beta Diversity

Difference in the microbial composition between environments (including distance or dissimilarity metrices)

```
metadata_beta     <- "SampleSite"
permutations      <- 9999
sampledf_beta     <- data.frame(sample_data(phylotable_rare_otu))


dist.uni          <- distance(phylotable_rare_otu, method = "unifrac")

## Warning in UniFrac(physeq, ...): Randomly assigning root as -- denovo7528
## -- in the phylogenetic tree in the data you provided.

## Warning in matrix(tree$edge[order(tree$edge[, 1]), ][, 2], byrow = TRUE, :
## DatenlÃ¤nge [10461] ist kein Teiler oder Vielfaches der Anzahl der Zeilen
## [5231]


dist.wuni         <- distance(phylotable_rare_otu, method = "wunifrac")
## Warning in UniFrac(physeq, weighted = TRUE, ...): Randomly assigning root
## as -- denovo5293 -- in the phylogenetic tree in the data you provided.

## Warning in UniFrac(physeq, weighted = TRUE, ...): DatenlÃ¤nge [10461] ist
## kein Teiler oder Vielfaches der Anzahl der Zeilen [5231]


anosim.uni        <- with(sampledf_beta, anosim(dist.uni, sampledf_beta[,meta
data_beta], permutations = permutations, distance = "unifrac"))

anosim.wuni       <- with(sampledf_beta, anosim(dist.wuni,

sampledf_beta[,metadata_beta], permutations = permutations, distance = "wunif
rac"))

anosim.uni        <- with(sampledf_beta, anosim(dist.uni,
sampledf_beta[,metadata_beta], permutations = permutations, distance = "unifr
ac"))

anosim.wuni       <- with(sampledf_beta, anosim(dist.wuni,
sampledf_beta[,metadata_beta], permutations = permutations, distance = "wunif
rac"))

beta_P_values     <- data.frame(Metric = c("Unweighted_Unifrac",
"Weighted_Unifrac"), P_anosim = c(anosim.uni$signif[1], anosim.wuni$signif[1]
))
```

```r
## Holm-Correction

beta_P_values$holm_anosim <- p.adjust(beta_P_values$P_anosim,method = "holm")

beta_P_values

## Metric                    R_anosim   P_anosim   holm_anosim
## 1 Unweighted_Unifrac      0.3185216  1e-04      2e-04
## 2 Weighted_Unifrac        0.1673045  1e-04      2e-04
```

### (Holm-corrected) R- und p-values for ANOSIM

| Metric | R_anosim | P_anosim | Holm_anosim |
|---|---|---|---|
| Unweighted_Unifrac | 0.3185216 | p = 1e$^{-04}$ | p = 2e$^{-04}$ |
| Weighted_Unifrac | 0.1673045 | p = 1e$^{-04}$ | p = 2e$^{-04}$ |

### PCoa Plots weighted and unweighted unifrac

```r
ord.uni     <- ordinate(phylotable_rare_otu, method = "PCoA", distance = "uni
frac")

## Warning in UniFrac(physeq, ...): Randomly assigning root as -- denovo20879
## -- in the phylogenetic tree in the data you provided.

## Warning in matrix(tree$edge[order(tree$edge[, 1]), ][, 2], byrow = TRUE, :
## DatenlÃ¤nge [10461] ist kein Teiler oder Vielfaches der Anzahl der Zeilen
## [5231]

ord.wuni    <- ordinate(phylotable_rare_otu, method = "PCoA", distance = "wun
ifrac")

## Warning in UniFrac(physeq, weighted = TRUE, ...): Randomly assigning root
## as -- denovo24008 -- in the phylogenetic tree in the data you provided.

## Warning in UniFrac(physeq, weighted = TRUE, ...): DatenlÃ¤nge [10461] ist
## kein Teiler oder Vielfaches der Anzahl der Zeilen [5231]

p2_wuni     <- plot_ordination(phylotable_rare_otu, ord.wuni, color =
metadata_beta, shape=metadata_beta) +
theme_bw() + ggtitle("PCoA + weighted unifrac")+
geom_point(size = 2)+
theme(text = element_text(size = 10))+
guides(color=guide_legend("Sample Site"),shape=guide_legend("Sample Site")) +
scale_colour_manual(values = c("#009933","#999999", "#0000FF"))
```

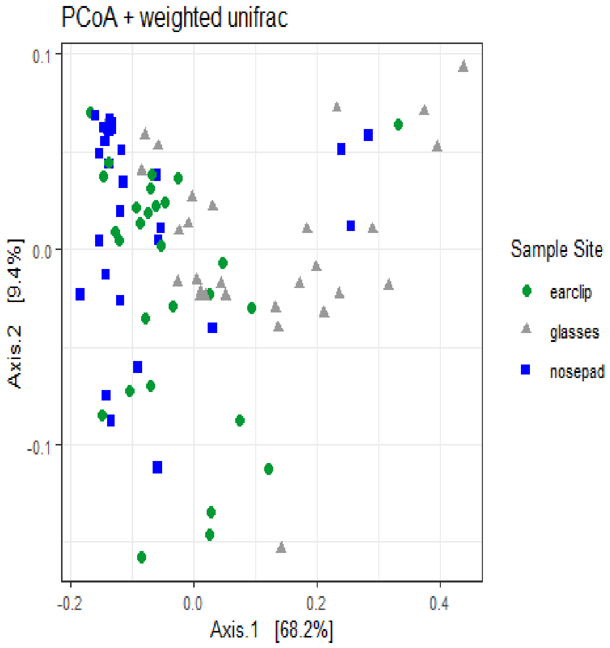


```
p2_uni      <- plot_ordination(phylotable_rare_otu, ord.uni,
color = metadata_beta, shape=metadata_beta) +
theme_bw() +
ggtitle("PCoA + unweighted unifrac")+
geom_point(size = 2)+
theme(text = element_text(size = 10))+
guides(color=guide_legend("Sample Site"), shape=guide_legend("Sample Site"))+
scale_colour_manual(values = c("#009933","#999999", "#0000FF"))

 p2_uni
```

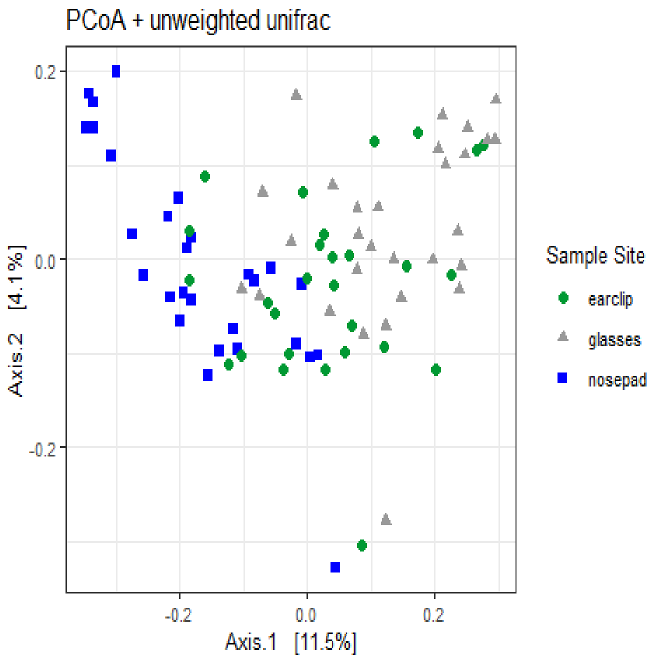

```r
sessionInfo()

## R version 3.5.2 (2018-12-20)
## Platform: x86_64-w64-mingw32/x64 (64-bit)
## Running under: Windows 10 x64 (build 18363)
##
## Matrix products: default
##
## locale:
## [1] LC_COLLATE=German_Germany.1252  LC_CTYPE=German_Germany.1252
## [3] LC_MONETARY=German_Germany.1252 LC_NUMERIC=C
## [5] LC_TIME=German_Germany.1252
##
## attached base packages:
## [1] stats     graphics  grDevices utils     datasets  methods   base
##
## other attached packages:
## [1] ape_5.3           coin_1.3-0        survival_2.44-1.1 vegan_2.5-5
## [5] lattice_0.20-38   permute_0.9-5     ggplot2_3.2.0     phyloseq_1.26.1
##
## loaded via a namespace (and not attached):
##  [1] zoo_1.8-6           modeltools_0.2-22   tidyselect_0.2.5
##  [4] xfun_0.8            reshape2_1.4.3      purrr_0.3.2
##  [7] splines_3.5.2       rhdf5_2.26.2        colorspace_1.4-1
## [10] htmltools_0.3.6     stats4_3.5.2        yaml_2.2.0
## [13] mgcv_1.8-28         rlang_0.4.0         pillar_1.4.2
## [16] withr_2.1.2         glue_1.3.1          BiocGenerics_0.28.0
## [19] multcomp_1.4-10     matrixStats_0.54.0  foreach_1.4.4
## [22] plyr_1.8.4          stringr_1.4.0       zlibbioc_1.28.0
## [25] Biostrings_2.50.2   munsell_0.5.0       gtable_0.3.0
## [28] mvtnorm_1.0-11      codetools_0.2-16    evaluate_0.14
## [31] labeling_0.3        Biobase_2.42.0      knitr_1.25
## [34] IRanges_2.16.0      biomformat_1.10.1   parallel_3.5.2
## [37] TH.data_1.0-10      Rcpp_1.0.1          scales_1.0.0
## [40] S4Vectors_0.20.1    jsonlite_1.6        XVector_0.22.0
## [43] digest_0.6.19       stringi_1.4.3       dplyr_0.8.3
## [46] grid_3.5.2          ade4_1.7-13         tools_3.5.2
## [49] sandwich_2.5-1      magrittr_1.5        lazyeval_0.2.2
## [52] tibble_2.1.3        cluster_2.1.0       crayon_1.3.4
## [55] pkgconfig_2.0.2     libcoin_1.0-4       MASS_7.3-51.4
## [58] Matrix_1.2-17       data.table_1.12.2   assertthat_0.2.1
## [61] rmarkdown_1.13      iterators_1.0.10    Rhdf5lib_1.4.3
## [64] R6_2.4.0            multtest_2.38.0     igraph_1.2.4.1
## [67] nlme_3.1-140        compiler_3.5.2
```

=============================================================================
